# Allele-specific genomics decodes gene targets and mechanisms of the non-coding genome

**DOI:** 10.1093/nar/gkaf912

**Published:** 2025-10-21

**Authors:** Tim P Hasenbein, Sarah Hoelzl, Stefan Engelhardt, Daniel Andergassen

**Affiliations:** Institute of Pharmacology and Toxicology, Technical University Munich (TUM), 80802 Munich, Germany; DZHK (German Centre for Cardiovascular Research), Partner Site Munich Heart Alliance, 80336 Munich, Germany; Institute of Pharmacology and Toxicology, Technical University Munich (TUM), 80802 Munich, Germany; DZHK (German Centre for Cardiovascular Research), Partner Site Munich Heart Alliance, 80336 Munich, Germany; Institute of Pharmacology and Toxicology, Technical University Munich (TUM), 80802 Munich, Germany; DZHK (German Centre for Cardiovascular Research), Partner Site Munich Heart Alliance, 80336 Munich, Germany; Institute of Pharmacology and Toxicology, Technical University Munich (TUM), 80802 Munich, Germany; DZHK (German Centre for Cardiovascular Research), Partner Site Munich Heart Alliance, 80336 Munich, Germany

## Abstract

A large proportion of disease variants is found in non-coding RNAs (ncRNAs), gene loci that have been identified as key regulatory elements. However, for most ncRNAs, their targets are unknown, hindering our ability to understand complex diseases. Here, we found that allele-specific ncRNAs were enriched nearby allelic protein-coding genes (pcGenes), suggesting that the allele-specific information could be used to predict *cis*-acting ncRNA-targets. We translated this concept into the Allelome.LINK framework and applied it to the major mouse organs, revealing 397 events where the allele-specific expression (ASE) of a ncRNA correlated or anticorrelated with the ASE of a nearby pcGene, suggesting either enhancing or repressive regulatory interactions. Integration of H3K27ac heart ChIP-seq enabled the linkage of putative allelic enhancers to allele-specific gene loci and provided insight into ncRNA- versus DNA-mediated regulatory effects. Next, we applied our strategy to the largest human dataset including tissues from nearly 1000 individuals. Given the high genetic diversity across humans, each individual allows for the discovery of novel ASE correlation events. We uncovered 2291 ncRNA-mRNA ASE events along with their mechanisms, which we benchmarked against sample-matched eQTLs, yielding a high validation rate of 77.47%. Further GWAS integration assigned variants overlapping informative ncRNA to their pcGene targets. As more sequencing data and risk variants become available, this strategy has the potential to decode the entire *cis*-acting landscape of the non-coding genome.

## Graphical Abstract

**Graphical Abstract:**
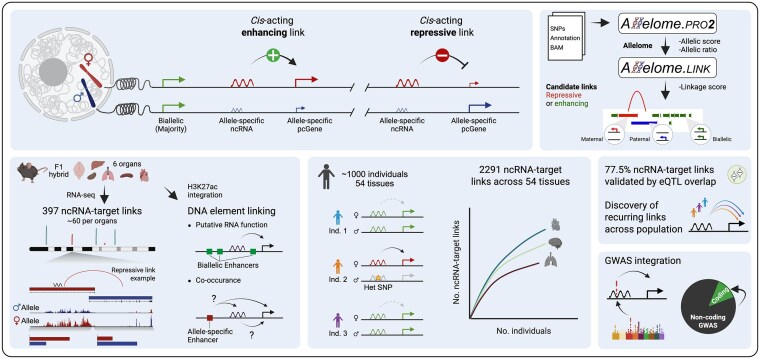

## Introduction

Only ∼1% of the human genome consists of protein-coding genes (pcGenes), leaving the majority as non-coding. These non-coding segments contain essential regulatory elements, including promoters, enhancers, and non-coding RNAs (ncRNAs) that determine the temporal and spatial activation or repression of genes. This process of transcriptional regulation is implicated in numerous human diseases, as the majority of disease-associated variants are located within non-coding regulatory regions and exert their effects through gene regulation [1, 2]. While regulatory elements across tissues and developmental stages have been extensively mapped [3–5], and genome-wide association studies (GWAS) have helped catalog human genetic disease variants [6], the specific target genes and mechanisms-of-action remain largely elusive. This knowledge gap hinders our ability to understand how variants within non-coding regulatory regions cause diseases, which is important for developing effective treatments.

Eighty percent of the human genome undergoes transcription, highlighting the prominence of ncRNAs within the non-coding genome [5, 7]. Recent database analysis indicates that a significant fraction of disease-associated variants overlaps with ncRNA loci, underscoring their potential significance in the context of diseases [8]. The most prominent subclass of ncRNAs is long non-coding RNAs (lncRNAs). Over the past decade, >60 000 lncRNAs have been identified in various human tissues [9], which has generated considerable interest in understanding their role in gene regulation. In contrast to pcGenes, ncRNAs display a remarkable degree of tissue specificity and much greater inter-individual expression variability among humans [10, 11]. However, their highly dynamic and context-specific expression limits the effectiveness of traditional approaches, such as expression quantitative trait loci (eQTL) mapping or co-expression analyses that require large sample sizes for identifying their targets [12, 13]. In addition, while recent perturbation-based screens provide functional insights, they are largely restricted to *in vitro* settings [14, 15]. To overcome these limitations, we present a novel approach that leverages allele-specific expression (ASE), a phenomenon in diploid organisms where one allele is expressed more than the other, to predict *cis*-acting ncRNA targets *in vivo* at the individual level. The rationale for linking ncRNAs to their target genes arises from studies of imprinted ncRNAs, which exhibit strong ASE and repress neighboring genes in *cis*, enabling the identification of their target genes through anti-correlating allelic patterns. Notable examples include *Airn* [16], *Meg3* [17], and the most extreme case, *Xist*, which represses an entire chromosome in *cis* [18]. Similarly, heterozygous knockout models that mimic ASE have revealed *cis*-acting, allele-specific regulatory effects on nearby genes, enabling the identification of potential targets [19, 20]. The vast majority of ASE is due to genetic differences between the alleles [21] that disrupt transcriptional regulation by impacting the binding of transcription factors to promoters or enhancers [22, 23], interfering with the function or activity of regulatory ncRNAs, or influencing allelic gene expression post-transcriptionally by affecting splice site selection or RNA stability [24]. As observed with imprinted ncRNAs, such patterns could enable the use of correlation and anti-correlating allelic expression to identify enhancing and repressive *cis*-acting ncRNA targets genome-wide.

Here, we found a significant clustering of allele-specific ncRNAs around allele-specific pcGenes in both mice and humans. Given the rarity of ASE, this suggested that the allele-specific information could be used to predict the target genes of ncRNAs. We translated this concept into a bioinformatics pipeline termed Allelome.LINK to identify correlating ncRNA-mRNA ASE events, enabling the prediction of the targets and regulatory mechanisms of *cis*-acting ncRNAs. Applying our strategy to major organs in mice and exploiting inter-individual variation in humans, we identified 397 and 2291 ncRNA-mRNA ASE events, respectively. Further integration of non-coding GWAS variants, linked a significant proportion of non-coding disease variants to their protein-coding targets. As more individual sequencing data and risk variants become available, this strategy will decode the functional landscape of the non-coding genome.

## Materials and methods

### Mouse strains

To generate F1 hybrids, C57BL/6J females (BL6, JAX: Strain #000664) were paired with CAST/EiJ males (CAST, JAX: Strain #000928). All mice were housed in an open-cage environment provided by the Technical University Munich Institute of Pharmacology and Toxicology. Organs were collected from wild-type F1 mouse strains in accordance with §4 of the German Animal Welfare Act (Tierschutzgesetz and Tierschutzversuchstierverordnung) and EU directive 2010/63.

### Tissue isolation and library preparation

Three replicates were harvested for brain, heart, liver, lung, kidney, and spleen from 9-week-old F1 hybrid mice (BL6 × CAST) and snap-frozen in liquid nitrogen. The GentleMACS Dissociator (program RNA_02_01) was used to homogenize tissues in 1 ml TRIzol per 50–100 mg sample. RNA isolation was conducted as described in the manufacturer’s instructions (Invitrogen, TRIzol Reagent, Cat. #15596018) using 1 ml of the sample solution. RNA-seq libraries were constructed with 100 ng of RNA and Illumina’s Stranded mRNA Prep Ligation Kit. The Agilent TapeStation System was used to assess the concentration and fragment length of the libraries. Sequencing was performed using a NovaSeq 6000 in 50 bp paired-end mode.

### Preprocessing of RNA-seq data

Paired-end RNA-seq data of 9-week-old mice were aligned to the GENCODE_M25GRCm38.p6_201911 primary assembly using STAR (v2.6.0c) [25]. Reads characterized by intron sizes exceeding 100 000, multimappers, and alignments containing non-canonical junctions were removed prior to subsequent analysis. Uniquely aligned reads were quantified using htseq-count and the *–*stranded reverse flag (HTSeq version 0.11.3) [26]. Transcripts per million (TPM) were calculated by custom R scripts for sample clustering based on Pearson correlation. Aligned bam files were further split into forward and reverse strands using a custom Perl script. BigWig files were generated using bam2wig.py. Public placental E12.5 RNA-seq data (2× CAST×FVB, 2× FVB×CAST) was downloaded from the GEO database for SRR3085950, SRR3085951, SRR3085952, SRR3085953 [22], as well as for the *Airn* knockout (SRR8753471, SRR8753472, SRR8753473, SRR8753474 SRR8753475, SRR8753476) [27].

### Allocation of the sequencing reads to the alleles using SNPsplit

Sequencing reads were further assigned to the parental alleles using SNPsplit v0.3.2 [28]. This process is independent of the Allelome.PRO2 run and serves solely for genome browser visualization. FASTQ files were merged across replicates per tissue to increase gene coverage. Subsequently, reads were mapped to an N-masked genome generated with *SNPsplit_genome_preparation* and STAR (v2.6.0c) for BL6/CAST single nucleotide polymorphisms (SNPs), which were obtained from the Sanger database (mgp.v5.merged.snps_all.dbSNP142.vcf) [29]. The same annotation file was used for alignment, but using the settings: *–alignIntronMax 100 000, –outFilterIntronMotifs RemoveNoncanonical*, *–outFilterMultimapNmax 1*, *–alignEndsType EndToEnd*, *–outSAMattributes NH HI NM MD*. The resulting aligned BAM file was separated by strand as described and used as input for SNPsplit v0.3.2.

### Generation of the Allelome.PRO2 pipeline

We developed Allelome.PRO2, an updated version of the previously published pipeline Allelome.PRO [30], to facilitate allele-specific analysis from individual samples rather than requiring data obtained from reciprocal crosses. This update enables its application to human and single-cell datasets and is compatible with diploid model organisms, provided that phased SNP data are available. The source code and a detailed manual on how to run Allelome.PRO2 are available at https://github.com/AndergassenLab/Allelome.LINK/. Briefly, as input Allelome.PRO2 requires an aligned sample file (BAM), annotation file (BED6) and a phased SNP file (BED4), similar to the original Allelome.PRO version. Along with these changes, we also removed the FDR-based mock comparison and the user-defined allelic ratio cut-off. A comprehensive log file has been implemented for tracking and debugging. As a result, Allelome.PRO2 reports, for every locus in the annotation file, the allelic ratios, allelic scores, and total SNP-overlapping reads. This enables the pipeline to be run on individual samples and allows users to apply custom filtering to the output file for allele-specific features.

### Development of the Allelome.LINK extension tool

To streamline the prediction of ncRNA-targets and their mode-of-action, we developed Allelome.LINK as an extension of Allelome.PRO2. Utilizing allele-specific data, this tool connects ASE loci in *cis* within user-defined genomic windows. As input, Allelome.LINK requires a text file formatted as the locus_table.txt output of Allelome.PRO2. The tool then filters loci with ASE values exceeding the specified cut-off threshold. Biased loci are intersected and linked if they co-occur within the defined genomic window. Mode-of-action is determined as enhancing or repressive based on allelic correlation or anti-correlation. A score is calculated for each linkage using the formula:


\begin{eqnarray*}
{\rm LS} = \;{\log _{10}}(\mathop {\min }\limits_{\left| {{\rm A}{{\rm S}_1}} \right|,\left| {{\rm A}{{\rm S}_2}} \right|} + \;1)\; \times \;\left( {1 - \left| {{\mathrm{\Delta }}{\rm AR}} \right|} \right),
\end{eqnarray*}

where AS is the allelic score for each locus as calculated by Allelome.PRO2 and ΔAR the differences in the allelic ratio calculated as


\begin{eqnarray*}
{\mathrm{\Delta AR}} = \left| {{\rm A}{{\rm R}_1} - 0.5} \right| - \left| {{\rm A}{{\rm R}_2} - 0.5} \right|.
\end{eqnarray*}

This definition is based on the assumption that regulatory genes correlate in their allelic bias. Each allelic ratio is standardized by subtracting 0.5 to center the values. Consequently, a small ΔAR signifies closely matched allelic ratios, irrespective of whether the bias is toward identical or opposite alleles. The allelic score is further implemented in the scoring calculation, assigning higher linkage scores to interactions characterized by robust allelic biases. This scoring accounts for the statistical significance derived from the *P*-value of a binomial distribution as described in [30]. As output, Allelome.LINK provides a linkage table ranked by linkage scores as well as BEDPE and BED files for intuitive visualization via genome browsers. Enhancing interactions are highlighted in green, while repressive predictions are marked in red. Additionally, each analysis run generates a comprehensive log file. By default, Allelome.LINK links all informative allele-specific loci regardless of biotype, including ncRNA-ncRNA and pcGene-pcGene interactions. This flexible design allows users to filter results according to their specific biological questions, ensuring broad applicability of the tool across diverse research contexts. Further information about Allelome.LINK and the source code is available at https://github.com/AndergassenLab/Allelome.LINK/.

### Allele-specific analysis of mouse samples

Allele-specific analysis for RNA-seq data was performed using the Allelome.PRO2 pipeline, an updated version of our previously published pipeline [30]. We used the RefSeq gene annotation, which was further split into forward and reverse strand [31]. To analyze the transcriptomic bodymap of F1 mice, we obtained 20 635 313 SNPs between C57BL_6NJ × CAST from the Sanger database (mgp.v5.merged.snps_all.dbSNP142.vcf) [29]. Allelome.PRO2 was run individually for each strand per sample, with a minimum read cut-off ≥1 per SNP. Strands were merged before pooling individual replicates. We retained loci with ≥20 total reads per gene in all three replicates. Reads and allelic ratios were summarized using median values, while the minimum value was selected for the allelic score to ensure robust allele-specific calling. Genes located on the X chromosome were removed prior to subsequent analyses in order to mitigate allele-specific bias due to skewed X-inactivation. To get the allele-specific information of X-linked genes expressed in the placenta, we used the publicly available data from [22] and followed the described processing steps with the exception of using a SNP file generated from mgp.v5.merged.snps_all.dbSNP142.vcf for CAST × FVB (*n* = 20 581 027). Replicates from forward and reverse crosses were pooled. Due to the lack of strand-specific information, unstranded files were used for the *Airn* knockout data, along with previously published CAST/FVB SNP file, which included 16 988 479 variants [27]. Replicates were pooled as described, using a total read count threshold ≥10.

### Linking ncRNA-mRNA ASE events using Allelome.LINK in mice

To link ncRNAs to their putative pcGene targets in mice, we used the mapped allelome of adult organs (spleen, kidney, lung, heart, liver, and brain) obtained from F1 hybrid mice. We applied Allelome.LINK using a total read cut-off ≥20 and an allelic ratio cut-off ≥0.7 or ≤0.3. The genomic window was set to ±100 kb due to the significant enrichment observed within this range. Subsequently, we filtered for non-coding to protein-coding interactions for further downstream analysis. For the prediction of *Xist* targets, we used an allelic bias ≥0.7 or ≤0.3 along with a chromosome-wide window. The genomic range was also adjusted for the *Airn* knockout data to ±4000 kb to encompass the entire *Airn/Igf2r* cluster. Allelic bias criteria were maintained at 0.7/0.3.

### Linking allele-specific enhancer marks to potential targets in mice

To investigate allele-specific enhancer regulation, we used publicly available H3K27ac ChIP-seq data derived from 2-month-old F1 hybrid mouse hearts (BL6 × CAST; ENCODE accession numbers: ENCFF344LBG, ENCFF530JVC) [7]. Peaks were called using the MACS2 callpeak function with the –broad parameter [32]. The peaks from both replicates were intersected and filtered for overlap with distal enhancer-like elements defined by ENCODE annotations [5]. Allele-specific enhancer activity was identified using Allelome.PRO2 with a total read cut-off ≥20 and an allelic ratio cut-off ≥0.7 or ≤0.3, using the MACS2 peak set as the annotation file. To infer regulatory interactions with gene loci, the allele-specific enhancer output was merged between replicates (mean allelic ratio) and combined with allele-specific RNA-seq data from the mouse heart. We then applied Allelome.LINK to this integrated dataset. The resulting set of enhancer-gene linkages was subsequently filtered to retain only enhancing DNA-RNA linkages for downstream analysis.

### Linking ncRNA-mRNA ASE events using human samples of the GTEx database

Human allele-specific measurements were derived from publicly available data generated by Castel *et al.* from the Genotype-Tissue Expression (GTEx) v8 release (phASER_WASP_GTEx_v8_matrix.gw_phased.txt) [33]. This large dataset contains 153 million allele-specific haplotype measurements across 15 253 samples from 54 human tissues. The haplotype data were merged with the sample information (GTEx_Analysis_v8_Annotations_SampleAttributesDS.txt) and formatted using R to match the Allelome.PRO2 output. Because the GTEx data lack strand-specificity, we filtered out ncRNAs with a combined exon length ≤200 bp and overlapping gene loci. To ensure data consistency, we used the GENCODE v26 annotation to match the annotation of the resource. After filtering the annotation, we maintained 21 921 loci, including 14 056 ncRNAs and 7865 pcGenes. A gene was classified informative with a total read cut-off ≥20 in at least one sample. Subsequently, we ran Allelome.LINK for each individual using an allelic ratio cut-off ≥0.7 or ≤0.3 and a window size of ±100 kb.

### Validation of linkages using eQTL information

To validate our predictions of ncRNA interactions with target genes, we obtained fine-mapped eQTL data from the GTEx v8 release matching the human haplotype measurements (GTEx_v8_finemapping_DAPG.txt) [34]. This dataset contains 21 648 584 fine-mapped eQTLs across 49 tissues. We extracted positional information and target gene names, resulting in a subset of 21 412 255 eQTLs across tissues, comprising 2 740 212 unique eQTLs. We then overlapped the positions of the eQTLs with the linked ncRNA loci and classified a linkage as confirmed if an overlapping eQTL was predicted to regulate the same target gene as predicted for the ncRNA.

### Intersecting GWAS-SNPs with linked ncRNAs

To elucidate the functional roles of non-coding GWAS variants, we downloaded all associations from the NHGRI-EBI GWAS Catalog (gwas_catalog_v1.0-associations_e110_r2023-10-11.tsv) [35]. This dataset comprised the SNP information for 132 201 unique variants. After the removal of SNP × SNP interactions and non-mappable variants, we filtered the GWAS Catalog data to include only SNPs associated with traits at genome-wide significance (*P*-value < 5 × 10^−8^, *n* = 89 469). We then overlapped these SNPs with the linked gene loci to identify GWAS-SNPs that are located within ncRNAs, pcGenes, or both interaction partner of the predicted linkage.

## Results

### Allele-specific ncRNAs are significantly enriched in proximity to allele-specific pcGenes

**Figure 1.**
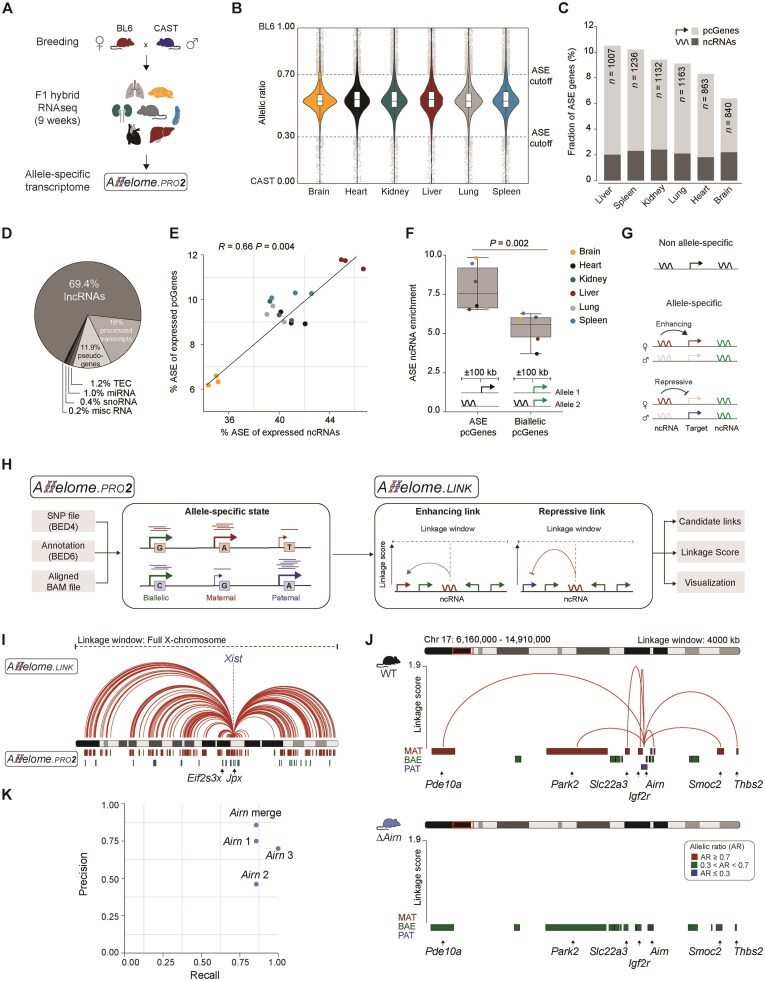
The Allelome.LINK strategy predicts mechanisms and targets of ncRNAs. (**A**) Schematic overview of the allele-specific mapping process. Using Allelome.PRO2, we mapped the allele-specific transcriptome of RNA-sequencing data acquired from F1 hybrid mice (BL6 × CAST). Tissue samples were collected from the brain, heart, lung, liver, kidney, and spleen (*n* = 3) of 9-week-old animals. (**B**) Violin plots showing the median allelic ratio of all replicates per tissue for autosomal genes. An allelic ratio of 1 indicates the BL6 allele, while 0 corresponds to the CAST allele. The dotted lines represent the defined cut-off values for ASE (total reads ≥20, allelic ratio ≥0.7 or ≤0.3). Boundaries of boxplots indicate the interquartile range around the median. Whiskers range from maximum to minimum values. Dots show gene loci with ASE. (**C**) Bar chart illustrating the proportions of allele-specific genes per tissue. Light gray denotes the percentage of pcGenes, while dark gray represents ncRNAs. (**D**) Pie chart showing biotype proportions for the allele-specifically expressed non-coding genes with available biotype information (*n* = 520, 52.74%). TEC: to be experimentally confirmed; misc RNA: miscellaneous RNAs that cannot be classified. (**E**) Correlation plot displaying the fraction of allele-specific protein-coding and non-coding genes normalized by the total number of protein-coding and non-coding loci, respectively. The color code represents the different tissue samples. The correlation was calculated using a Spearman correlation (*R* = 0.66, *P* = .004). (**F**) Box plot illustrating the percentage of allele-specific ncRNAs nearby allele-specific or biallelic pcGenes (±100 kb distance). Boundaries of the box indicate the interquartile range around the median. Whiskers range from maximum to minimum values. A Wilcoxon test was used to compare the allele-specific ncRNA enrichment around allele-specific and biallelic pcGenes (*P* = .002). (**G**) Overview of the allele-specific strategy for predicting target genes of ncRNA loci based on allele-specific correlation. NcRNAs were classified as repressive or enhancing depending on their expression bias relative to nearby targets. (**H**) Schematic overview of the Allelome.PRO2/LINK pipeline. The workflow starts by using Allelome.PRO2 to classify ASE based on sequencing reads overlapping heterozygous SNPs. Allelome.PRO2 outputs the allelic ratio and score for each locus, which can be used by Allelome.LINK. Subsequently, Allelome.LINK connects allele-specific loci within a user-defined genomic linkage window as either enhancing (green) or repressive (red) based on allelic correlation or anti-correlation providing candidate associations with a linkage score. Both pipelines also provide Integrative Genomics Viewer-compatible visualization files to support visual inspection. The color of the genes indicates their allele-specific status as biallelic (green), maternal (red), or paternal expression (blue). (**I**) Output of the Allelome.LINK pipeline for the lncRNA *Xist* of publicly available RNA-sequencing data from the placenta of E12.5 F1 hybrids (CAST × FVB *n* = 2, FVB × CAST *n* = 2). Red lines represent repressive interactions with pcGenes (total reads ≥20, allelic ratio >0.7 and <0.3, window size: full chromosome). Below the X chromosome, red lines indicate maternally expressed, while green lines show biallelic genes, including the known escape genes *Eif2s3x* and *Jpx*. (**J**) Allelome.LINK output of the *Igf2r*/*Airn* locus from placentas isolated from E12.5 embryos (*n* = 3 per genotype). The upper browser track shows the repressive targets of *Airn* and their allelic ratio as depicted by the color code in the wild-type (*n* = 3, median). The arcs represent repressive interactions between *Airn* and target genes while the height indicates the linkage score. The lower panel illustrates the results for the *Airn* promoter deletion (*n* = 3, median; total reads ≥10, allelic ratio ≥ 0.7 or ≤ 0.3; window size: 4000 kb). (**K**) Precision-recall plot evaluating the Allelome.LINK pipeline for *Airn* and the *Igf2r* locus. Precision and recall were calculated for individual replicates and combined.

To investigate the correlation between the number of allele-specific ncRNAs and the number of allele-specifically pcGenes, we first comprehensively mapped the allele-specific transcriptome of 9-week-old female F1 hybrid mice, generated by crossing BL6 females with CAST males (see “Materials and methods” section). This analysis encompassed six primary mouse organs: brain, heart, lung, liver, kidney, and spleen (replicates *n* = 3; Fig. 1A, Supplementary Fig. S1A, and Supplementary Table S1). Only high-confidence loci informative in all replicates were retained for downstream analysis. Genes with an allelic ratio ≥0.7 or ≤0.3 were classified as allele-specific (Fig. 1B). On average, 8.98% (*n* = 1040) of the informative genes showed ASE per tissue, ranging from 10.5% (liver, *n* = 1007) to 6.4% (brain, *n* = 840; Fig. 1C and Supplementary Table S2). Among these, a mean of 2.13% were ncRNAs, mainly corresponding to lncRNAs (69.4%; Fig. 1D). It should be noted that the RNA-seq approach used in this study enriches for polyadenylated transcripts, biasing detection toward longer ncRNAs and excluding non-polyadenylated species. Next, we compared the abundance of ncRNAs and pcGenes with an allelic bias across all tissues and found a positive correlation between allele-specific ncRNA proportions and allele-specific pcGenes (Spearman correlation: *R* = 0.66, *P* = .004, Fig. 1E) consistent with previous observations [22]. To investigate whether the allele-specific ncRNAs were significantly enriched near allele-specific pcGenes, suggesting that they might be co-regulated, we quantified their co-occurrence within various window sizes (Supplementary Fig. S1B). As a control, we calculated the enrichment of allele-specific ncRNAs to biallelic pcGenes. This analysis revealed a significant enrichment of allele-specific ncRNAs around allele-specific pcGenes up to ±100 kb distance (Wilcox test, *P* = .002; Fig. 1F and Supplementary Table S3), which indicates a co-regulatory association between them. This further suggests that the allele-specific information can be exploited to predict the *cis*-targets of ncRNAs. In addition, the underlying regulatory mechanism may be inferred based on the expression pattern: a repressive interaction when both the ncRNA and the nearby pcGene show expression bias toward opposite alleles, and an enhancing interaction when there is a correlative bias from the same allele (Fig. 1G).

### The Allelome.LINK strategy correctly assigns targets and mechanisms of imprinted lncRNAs

To facilitate the prediction of ncRNA-targets and their mode-of-action, we adapted the Allelome.PRO pipeline [30] to make it more user-friendly and usable to multiple applications including the identification of allele-specific genomic features from individual samples or from a single cell (see “Materials and methods” section). As an extension to Allelome.PRO2, we developed the Allelome.LINK pipeline (Supplementary Fig. S2A and B). This was designed to use the allele-specific information to identify allele-specific loci within user-defined windows and predict enhancing or suppressing *cis*-effects based on allelic bias towards identical or opposite alleles, respectively (Fig. 1H). We first tested our pipeline by predicting the regulatory targets of the extensively studied lncRNA *Xist*, which epigenetically silences specifically the paternal X chromosome in extraembryonic lineages [36, 37]. Using this system, we aimed to predict the targets in the placenta and accurately identified *Xist* as a repressive lncRNA for most informative X-linked genes (87.41%; Fig. 1I and Supplementary Table S4). Notably, the pipeline did not assign repressive associations to the known escape genes *Eif2s3x* and *Jpx* [38, 39], highlighting the robustness of our approach. We performed further validation by testing the predictions for known targets of the paternally expressed lncRNA *Airn*, which silences genes within the *Igf2r*/*Airn* cluster in a *cis*-dependent manner [27, 40–42]. Using wild-type and *Airn* knockout RNA-seq datasets from F1 placentas [22, 43], we identified maternal expression for known *Airn* targets and confirmed biallelic expression in *Airn* knockouts (Fig. 1J). Importantly, Allelome.LINK correctly assigned *Airn* as a repressive ncRNA in the wild-type, with no linkages detected in the knockout model (Fig. 1J and Supplementary Table S5). Precision and recall were calculated separately for each replicate and for pooled samples using various read cut-offs (Supplementary Fig. S2C). The highest precision (85.7%) was obtained using a read cut-off of ≥10 and merging replicates, while the recall remained above 85% (Fig. 1K). Taken together, these showcases highlight the potential of the Allelome.LINK approach in predicting the *cis*-targets and mode-of-action of regulatory ncRNAs.

### Allele-specific genomics assigns targets and mechanisms to a significant fraction of ncRNAs across mouse organs

**Figure 2.**
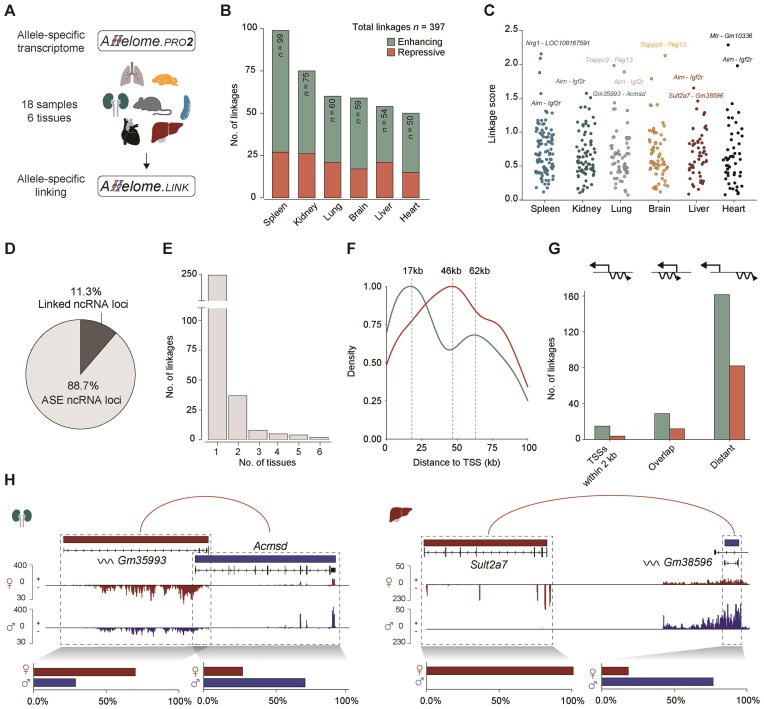
Allelome.LINK elucidates shared and tissue-specific ncRNA-to-target links and their mode-of-action. (**A**) Schematic overview of the Allelome.LINK workflow in mice. Candidate linkages were predicted using the allele-specific transcriptome data of the brain, heart, lung, liver, kidney, and spleen. (**B**) Number of linkages identified per tissue. Green denotes the enhancing interactions, while red represents repressive linkages. The numbers display the total abundance of linkages per tissue. (**C**) Manhattan plot showing the distribution of linkages per tissue along the linkage score. (**D**) Pie chart displaying the mean proportion of allele-specific ncRNA loci across tissue. (**E**) The number of shared linkages across the six different tissues. (**F**) Density plot showing the distribution of TSS distances between ncRNAs and target genes. Enhancing (green) and repressive (red) interactions are plotted separately. Dashed lines indicate the distance at which each interaction type reaches peak density. (**G**) Bar plots displaying the number of unique enhancing (green) and repressive (red) interactions across all samples, grouped by category: interactions where both TSSs are within 2 kb, where the genes overlap, or where they are distantly located. (**H**) Examples of two high-confident linkages displayed in the Integrative Genomics Viewer browser. The upper part shows the Allelome.LINK output, with red arcs representing repressive linkages. Gene colors indicate the allele-specific ratio towards maternal (red) and paternal (blue) expression. The panel below shows strand- and allele-specific mapping of sequencing reads for the maternal and paternal allele using SNPsplit [28]. Bar plots quantify mapped reads for each allele. Left: Repressive anti-sense linkage between *Gm35993* and *Acmsd*, detected in the kidney. Right: Intergenic linkage identified in the liver, where *Gm38596* is predicted to suppress *Sult2a7*.

After validation, we applied the Allelome.LINK approach to the generated allele-specific mouse bodymap (Fig. 2A). On average, we predicted 66.2 linkages per tissue, with the spleen showing the highest number of ncRNA-mRNA ASE events (*n* = 99). Notably, there was a higher number of predicted enhancing than repressive linkages across all organs (270 out of 397; binomial test *P* = 2.9 × 10^-13^, Fig. 2B). Among the enhancing linkages, 82.53% of ncRNAs had only one target, with a similar pattern observed for repressive linkages (83.34%, Supplementary Fig. S2E), suggesting that the enrichment of enhancing interactions is not driven by a small subset of highly connected ncRNAs. The well-established imprinted interaction between *Airn* and *Igf2r* was one of the top linkages in all tissues except the brain, confirming previous findings and further validating our strategy [22] (Fig. 2C). In total, the Allelome.LINK approach successfully linked an average of 11.3% of the allelic ncRNAs to their putative target genes (Fig. 2D). Notably, the majority of linkages were tissue-specific (81.52%, *n* = 247), while only 18.48% (*n* = 56) were observed in two or more tissues (Fig. 2E). Interestingly, while repressive linkages showed an evenly distributed target distance, with the highest peak observed around 46 kb, enhancing linkages predominantly targeted nearby genes, peaking at 17 kb, suggesting potential regulation via shared promoters (Fig. 2F). Indeed, within this peak range, 15 out of 45 predicted enhancing ncRNA targets have transcription start sites (TSSs) within a ±2 kb defined promoter region [44], supporting the involvement of shared promoters (Fig. 2G). We selected two examples of high-confident linkages to highlight the target gene prediction based on the allelic read counts. First, a repressive anti-sense link between the ncRNA *Gm35993* and the pcGene *Acmsd* detected in the kidney (Fig. 2H, left panel), and second, an intergenic repressive link identified in the liver, where *Gm38596* is predicted to suppress *Sult2a7* (Fig. 2H, right panel).

**Figure 3.**
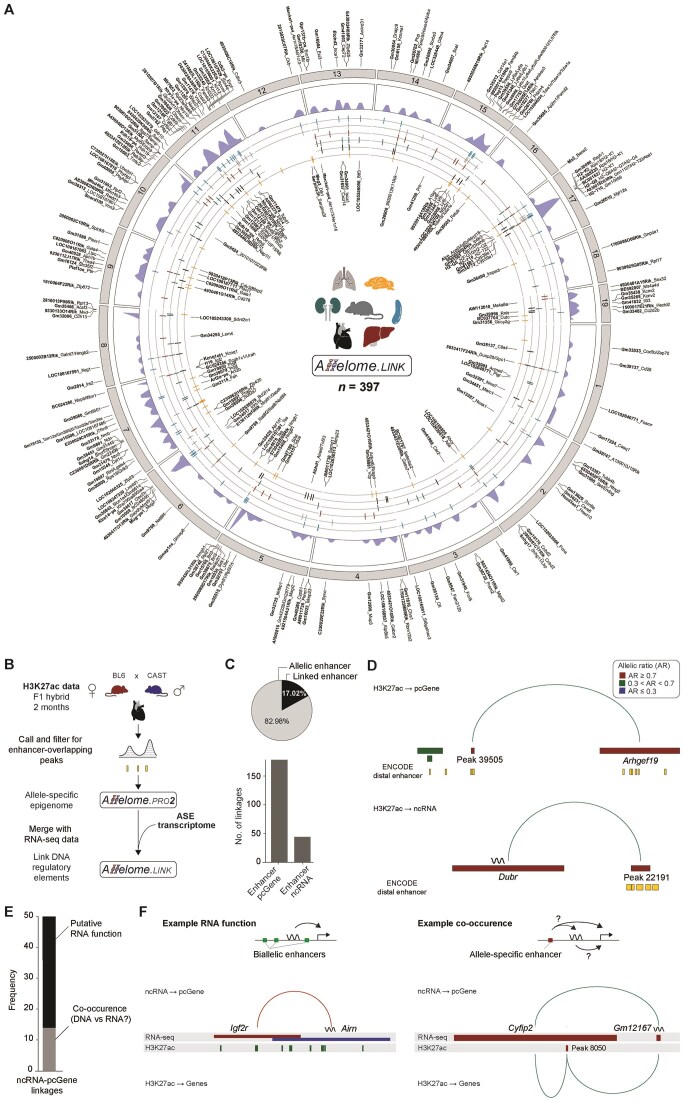
Overview of candidate ncRNA-to-target linkages across diverse mouse tissues and their inferred mode-of-action in the heart. (**A**) Candidate interactions are presented in a chord plot spanning chromosome 1–19. Linkages on the outside indicate enhancing interactions, while internal linkages indicate repressive links. For each linkage, the ncRNA is highlighted in bold font and the target is separated by an underscore. For ncRNAs with multiple targets, the pcGenes are separated by dashes. Density plots show the distribution of linkages across chromosomes. The genomic location of the ncRNA is shown in the inner circle, with each line corresponding to a tissue. (**B**) Schematic overview of the Allelome.LINK H3K27ac-analysis in the mouse heart. H3K27ac ChIP-seq data from 2-month-old BL6 × CAST F1 hybrid mouse hearts were used to identify allele-specific enhancer peaks. The allele-specific enhancer data were then integrated with allelic transcriptome data and analyzed using Allelome.LINK to infer enhancer-gene relationships. (**C**) The pie chart illustrates the proportion of allele-specific enhancer peaks assigned to target genes. The bar graph shows the number of enhancer linkages to pcGenes (*n* = 178) and ncRNAs (*n* = 44). (**D**) Representative examples of enhancer-gene linkages inferred from the allele-specific mapping. Top: the enhancer Peak 39505 was linked to the pcGene *Arhgef19*. Bottom: the enhancer Peak 22191 was linked to the ncRNA *Dubr*. Arcs depict enhancer-target relationships, while gene body color indicates the allele-specific activity for the parental allele (red: maternal, green: biallelic). (**E**) Classification of the 50 ncRNA-pcGene correlation events identified in the heart by their association with enhancer elements. Gray indicates co-occurence of allele-specific enhancers, while black indicates the lack of nearby allelic enhancer marks, suggesting ncRNA-mediated regulatory roles. (**F**) Example loci with putative direct ncRNA function include the *Airn-Igf2r* locus, which is covered only by biallelic H3K27ac peaks (left), and an example of co-occurrence with a nearby allelic enhancer, where a maternal H3K27ac peak overlaps the *Cyfip2* gene (right).

Overall, our analysis yielded a total of 397 predicted ncRNA-mRNA ASE events across all tissues (Fig. 3A and Supplementary Table S6). While we focus on ncRNA-pcGene pairs, it is also plausible that the reverse occurs, whereby pcGenes can regulate nearby ncRNAs or other pcGenes [19]. Thus, by default, Allelome.LINK identifies all combinations of allele-specific interactions, including pcGene-pcGene and ncRNA-ncRNA links (Supplementary Fig. S2D and Supplementary Table S7).

To investigate whether the ASE correlation between ncRNAs and their targets arises from ncRNA-mediated mechanisms or from enhancers that independently regulate both, we integrated publicly available H3K27ac ChIP-seq data from age- and strain-matched F1 mouse hearts. Using the Allelome.PRO2 pipeline, we identified 1187 allele-specific H3K27ac peaks out of 13 209 informative peaks (see “Materials and methods” section), which were used as input to Allelome.LINK along with the allele-specific heart transcriptome to link enhancer marks to their associated ncRNAs or pcGenes (Fig. 3B). This analysis linked 17.02% of the allele-specific enhancer marks to nearby genes, yielding 222 putative enhancer-gene linkages, including 178 pcGenes and 44 ncRNAs (Fig. 3C and D). We then used these enhancer links to interpret the 50 ncRNA-mRNA correlation pairs detected in the mouse heart (Fig. 3E and F). For most correlations (72%, *n* = 36), no nearby allele-specific enhancer marks were found, implying that the ncRNA itself may exert a regulatory effect (Fig. 3E and Supplementary Table S8). In the remaining 14 cases, the correlated gene and ncRNA coincided with a nearby allele-specific enhancer mark, raising the possibility of co-regulation via shared DNA elements. Notably, this analysis cannot exclude the possibility that the promoter itself functions as an enhancer, as reported previously [19, 45]. Nevertheless, the analysis showcases the utility of Allelome.LINK in linking DNA regulatory elements to their associated targets and in shedding light on underlying regulatory mechanisms. To facilitate further exploration and validation of the predicted associations, we have made all candidate links available for visual inspection, providing a valuable resource for the research community (see “Data availability” section).

### Exploiting inter-individual variation in humans elucidates targets and mechanisms of ncRNAs across tissues

**Figure 4.**
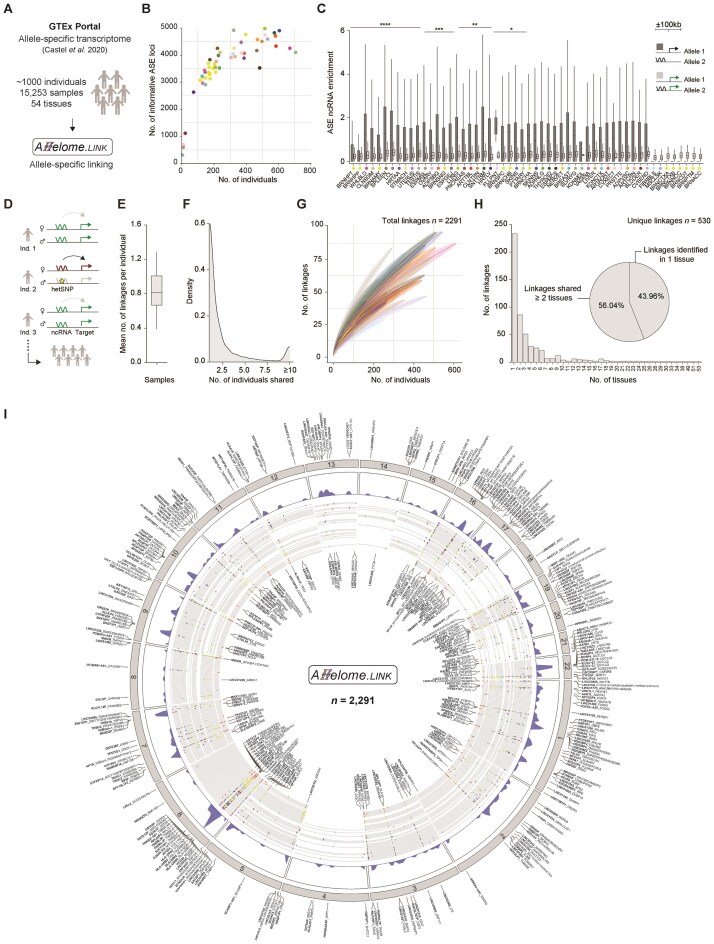
Applying the Allelome.LINK approach to the GTEx database predicts 2291 ncRNA-mRNA ASE events in humans. (**A**) Schematic overview of predicting human ncRNA-mRNA ASE events using the GTEx database. Allelome.LINK was applied to the publicly available data generated by Castel *et al.* (GTEx v8 release) [33]. This dataset contains 153 million allele-specific haplotype measurements across 15 253 samples and 54 different human tissues. (**B**) Scatter plot showing the number of individuals along the number of unique allele-specific loci after removing ncRNAs ≤200 bp in exon length and overlapping genes. Informative loci (total read number ≥20) were considered allele-specific with allelic bias ≥0.7 or ≤0.3 *(n* = 193 327). The color code represents the different sampling sites. (**C**) Enrichment of allele-specific ncRNAs around pcGenes. Box plots show the proportions of allele-specific ncRNAs around allele-specific (dark gray) and biallelic (light gray) pcGenes within ±100 kb distance, across individuals per tissue. Boundaries of the box indicate the interquartile range around the median. Whiskers range from maximum to minimum values. Wilcoxon tests were used to compare allele-specific ncRNA enrichment around allele-specific and biallelic genes. The number of asterisks indicates the significance level (*P*-value < .05). (**D**) Schematic overview demonstrating how genetic diversity in humans leads to the discovery of novel linkages. While the enhancing interaction is evident in all three individuals, only individual 2 allows the detection of the association due to a heterozygous SNP that induces ASE of the ncRNA. In individuals 1 and 3, this interaction is masked by the biallelic expression of the ncRNA. (**E**) Mean number of linkages per individual across all tissues. Boundaries of the box indicate the interquartile range around the median. Whiskers range from maximum to minimum values. (**F**) Density plot illustrating the number of individuals that share a linkage. (**G**) Line plot showing the mean number of linkages for each tissue, along with the number of individuals. Means and standard deviations were calculated by random sampling with 1000 iterations. (**H**) Bar plot illustrating the number of linkages that are shared across a diverse number of tissues. The pie plot represents the proportions of the 530 unique linkages that are tissue-specific and shared in ≥2 tissues. (**I**) Chord plot of human candidate interactions spanning chromosomes 1–22. Linkages on the outside of the chord plot indicate interactions that were detected as enhancing across most samples, while internal linkages indicate repressive links. For each interaction, the ncRNA is highlighted in bold font, and the target is separated by an underscore. Density plots show the distribution of linkages across chromosomes. The genomic location of the ncRNA is shown in the inner circle, with each line corresponding to a tissue according to the color code.

To extend the approach beyond mouse models, we applied our linking strategy to allele-specific haplotype data of the GTEx data base [33]. This comprehensive dataset includes 153 million haplotype measurements across 15 253 samples and 54 human tissues of nearly 1000 individuals (Fig. 4A). As the GTEx data are not strand-specific, we removed gene loci with overlapping exons to mitigate the allelic bias of overlapping transcripts. This process yielded 1825 informative ncRNA and 6281 pcGene loci, comprising 924 440 and 27 155 698 allele-specific measurements, respectively (*n* = 8106 loci, total read cut-off ≥20; Supplementary Fig. S3). On average, we detected 3580 (ncRNAs *n* = 312, pcGenes *n* = 3268) unique loci with ASE per sampling site. Generally, we found that the number of allele-specific loci increased with the number of individuals (Fig. 4B and Supplementary Table S9). For instance, the Kidney-Medulla showed the lowest number of ASE genes (*n* = 310; four individuals), while the lung showed the highest number (*n* = 4983; 515 individuals).

To validate the non-random association between allele-specific ncRNAs and proximal allelic pcGenes in humans, we quantified their co-occurrence within a distance of ±100 kb per individual and tissue. In line with findings in mice, we observed a significant enrichment of allele-specific ncRNAs in the proximity of allelic pcGenes for half of the investigated tissues (*n* = 27 of 54, *P*-value < .05, Fig. 4C, Supplementary Table S10). Hence, we proceeded to leverage the Allelome.LINK approach on the extensive allele-specific dataset, allowing us to predict human ncRNA-mRNA ASE events. Given the outbred nature of humans, each individual is expected to have a unique allele-specific landscape shaped by their personalized set of genetic variants. This diversity provides an opportunity for the discovery of novel ncRNA-mRNA ASE events, with each individual potentially revealing further linkages. Notably, the ncRNA mechanisms and targets identified through these individual-specific linkages are expected to be common across individuals but can only be detected in individuals with a genotype that leads to allelic bias (Fig. 4D). On average, each individual revealed approximately one linkage per sampling site (Fig. 4E). The majority of the linkages (63.77%) were unique to a single individual, while 36.23% were detected in multiple samples (Fig. 4F and Supplementary Table S11). To test whether each individual contributes to new linkages and whether we already reached saturation in this large dataset, we generated a saturation curve for each tissue. Intriguingly, we observed that each sample contributes to identifying novel links, as we did not observe a saturation at any sampling site (Fig. 4G and Supplementary Table S12). This finding highlights the power of this approach and the future potential of adding more individuals to uncover new associations by exploiting the genetic variation inherent in the human population. Consistent with the findings in mice, our analysis revealed many tissue-specific linkages, accounting for 43.96% (*n =* 233), while 56.04% (*n* = 297) were shared across two or more tissues (Fig. 4H and Supplementary Table S13). These findings underscore the extensive tissue-specific regulatory roles of ncRNAs. Overall, we identified 2291 human ncRNA-mRNA ASE events across all tissues, comprising 324 unique linked ncRNAs (Fig. 4I and Supplementary Table S14). Hence, we predicted the *cis*-targets of 17.75% of the informative ncRNA loci using the allele-specific approach. This resource provides insight into the complex interplay between human ncRNAs and their targets and serves as an ideal starting point to select candidates based on target function (see “Data availability” section).

### The majority of identified human linkages are confirmed by eQTL data

**Figure 5.**
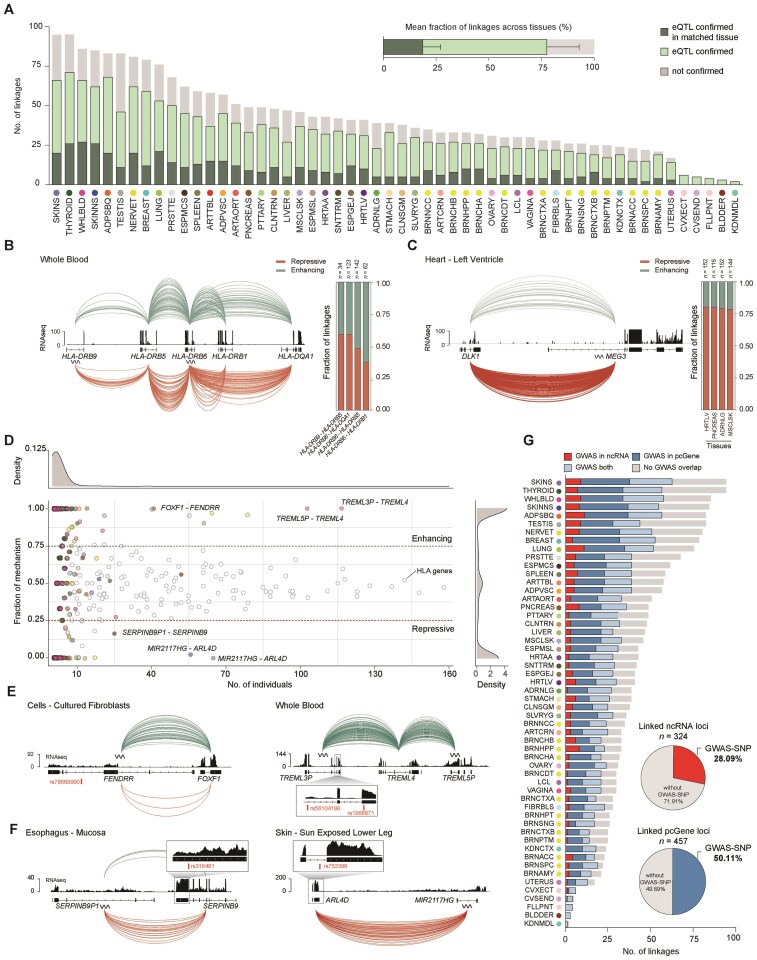
Validating the anticipated ncRNA-mRNA ASE events and linking non-coding GWAS-SNPs to their protein-coding targets. (**A**) Stacked bar chart illustrating the percentage of eQTL validation for the identified linkages per tissue. Linkages were classified as confirmed if an eQTL that overlaps the ncRNA gene body affects the expression of the linked target gene. Dark green represents the percentage of linkages confirmed by an eQTL within the same tissue, while light green represents the percentage of linkages validated by eQTLs identified in different tissues. The fraction of linkages that could not be confirmed by eQTLs is depicted in gray. Bar plot above showing the mean fraction of eQTL-validated linkages across all tissues (*n* = 49). Error bars indicate the standard deviation. (**B**) Allelome.LINK output for the HLA genes: *HLA-DRB9*, *HLA-DRB5*, *HLA-DRB6*, *HLA-DRB1*, *HLA-DQA1* in whole blood samples (*n* = 611). NcRNAs are indicated with a wavy line. Green arcs represent enhancing (*n* = 178), while red arcs indicate repressive linkages (*n* = 183). The height of the arcs is proportional to the linkage score. The RNA-sequencing track was obtained from GTEx and represents a single sample of the tissue. The bar plot illustrates the fraction of mechanism (green: enhancing, red: repressive) for each of the four linkages across individuals. (**C**) Allelome.LINK output as in panel (B) for *MEG3* and *DLK1* across Heart-Left Ventricle samples (*n* = 386). It is important to note that this interaction was excluded from our final dataset due to the filtering of gene loci with overlapping exons but serves as a robust example for the mechanism assignment. The bar plot illustrates the fraction of mechanism (green: enhancing, red: repressive) for the *MEG3-DLK1* interaction in the tissues: Heart-Left Ventricle, Pancreas, Adrenal gland, and Muscle-Skeletal. (**D**) Scatter plot showing the fraction of enhancing or repressive mechanisms along the number of individuals. The mechanism fraction is calculated by determining the number of individuals that show a specific linkage as enhancing, to assess consistency across samples within a tissue. 1 indicates all individuals show the linkage as enhancing, while 0 means all detections were repressive. The color code is according to the tissue. Dots without color represent linkages that include genes of the HLA cluster. Density plots summarize linkages across tissues, illustrating their distribution based on the number of individuals and the proportion of mechanisms observed. (**E**) Two examples of enhancing ncRNA-target links classified as high-confidence: *FENDRR-FOXF1* in cultured fibroblasts, and *TREML5P-TREML4* and *TREML3P-TREML4* in whole blood. The lncRNA *FENDRR* overlaps a GWAS-SNP located in its intron (left), while the ncRNA *TREML3P* overlaps both an intronic and exonic GWAS-SNP (right). NcRNAs are indicated with a wavy line. Zoom-outs highlight genome-wide significant GWAS variants (red, *P* < 5 × 10^−8^). (**F**) Two examples of repressive ncRNA-target links classified as high-confidence: *SERPINB9P1-SERPINB9* in esophagus mucosa, and *MIR2117HG-ARL4D* in sun-exposed skin of the lower leg. The pcGene *SERPINB9* overlaps an exonic GWAS-SNP (left), while the pcGene *ARL4D* overlaps an intronic GWAS variant (right). (**G**) Stacked bar chart illustrating the number of linkages, where either the ncRNA (red), the pcGene (blue), or both interacting partner (light blue) overlap genome-wide significant GWAS-SNPs (*P* < 5 × 10^−8^) per tissue. The upper pie chart illustrates the proportion of linked ncRNA loci that overlap GWAS-SNPs out of the total number of linked ncRNAs (*n* = 324). The lower pie chart shows the fraction of linked pcGenes that overlap significant GWAS-SNPs, from the total fraction of linked pcGenes (*n* = 457).

In order to confirm the predicted ncRNA-target gene associations, we overlapped the linked ncRNA loci with eQTL data that derived from the same samples, containing 21 412 255 fine-mapped eQTLs of 49 tissues [34]. eQTL data were absent for the Kidney-Medulla, Fallopian Tube, Cervix-Endocervix, Cervix-Ectocervix, and Bladder. We classified a linkage as confirmed if an overlapping eQTL was predicted to regulate the same target gene as predicted for the ncRNA. Remarkably, we confirmed an average of 77.47% (sd = 9.83) of the linkages with eQTLs across all 49 sampling sites (Fig. 5A). Of these eQTLs, a mean of 18.72% of our ncRNA-mRNA ASE events were confirmed in the corresponding tissue, with Cells-Cultured Fibroblasts showing the highest eQTL support of 32.14% (Supplementary Table S15). We then investigated whether the ncRNA and its predicted target gene co-localize within the same topologically associating domain (TAD), as such spatial proximity would support a functional regulatory relationship within a shared chromatin neighborhood. Consistent with this, we found that, on average, 78.2% of predicted linkages in the human dataset fall within the same TAD (Supplementary Fig. S4A and B, Supplementary Table S17). A similar trend was observed in mouse (81.7%; Supplementary Fig. S4C and Supplementary Table S18), further supporting the biological plausibility of our *cis*-regulatory predictions. In conclusion, this analysis validated a substantial proportion of the predicted linkages, supporting the reliability of our approach in assigning ncRNA loci to their potential targets.

### Discovery of high-confidence ncRNA linkages through over-representation of targets and mechanisms across individuals

To evaluate whether consistency in regulatory mechanism predictions can distinguish high-confidence from low-confidence linkages, we examined the proportion of enhancing and repressive assignments for linkages that replicate across individuals. True regulatory relationships are expected to show correlating ASE biases, leading to stable mechanism predictions, while inconsistent assignments suggest the absence of a functional regulatory relationship. As a negative control, we analyzed linkages involving genes in the highly polymorphic HLA cluster, which exhibit variable allelic statuses among individuals [46]. In whole blood samples, these linkages showed nearly equal proportions of enhancing (49.25%) and repressive (50.75%) assignments across individuals, suggesting no strong population-wide bias in the replication frequency of either mechanism (Fig. 5B). In contrast, we tested our strategy on the imprinted *MEG3*/*DLK1* loci, where the lncRNA *MEG3* is known for maternal-mediated *DLK1* repression [47, 48]. Although this interaction was filtered out from our final dataset due to the rigorous exclusion of gene loci with overlapping exons, this epigenetic interaction remains a robust example for assessing consistency across individuals, as its repressive interaction is independent of individual genotypes. We analyzed tissues where imprinting of *MEG3* and *DLK1* has been previously confirmed [49] and correctly identified 79.43% of 564 linkages as repressive (Fig. 5C), supporting the notion that a consistent mechanism assignment across individuals can serve as a useful indicator for identifying high-confidence regulatory linkages.

To elucidate high-confidence ncRNA-target linkages, we took advantage of the large number of individuals to determine how many links are represented in many individuals that consistently show repressive or enhancing mechanisms. Applying a consistency cut-off of ≥75%, requiring a linkage to be detected as either enhancing or repressive in at least 75% for a given tissue, we identified 35.0% repressive (*n* = 802) and 48.1% enhancing (*n* = 1102) linkages (Fig. 5D and Supplementary Tables S11 and S15). The mechanisms for the remaining predictions were randomly assigned (16.89%), with a significant proportion (36.69%) encompassing the HLA genes (Fig. 5D). Next, we investigated all linkages present in ≥10 individuals for a given tissue and identified 24 high-confident predictions (repressive *n* = 9, enhancing *n* = 15) that showed consistent mechanism prediction (≥75% enhancing or repressive). Among those linkages, we highlight the interaction between *FOXF1* and the lncRNA *FENDRR*, detected in 55 cultured fibroblast samples, with 52 consistently showing enhancing effects (Fig. 5D and E). This finding in humans is supported by a previous study in mice that identified a feedback loop between *Fendrr* and *Foxf1* [50, 51]. Another example is the pcGene *TREML4*, which is predicted to be positively associated with the two ncRNAs, *TREML3P* and *TREML5P*, supported by 117 and 103 enhancing interactions in whole blood, respectively (Fig. 5D and E). Additionally, we note the repressive linkage between the ncRNA *SERPINB9P1* and the target gene *SERPINB9* (*n =*21), as well as the ncRNA *MIR2117HG* and the pcGene *ARL4D* (*n =*65, Fig. 5D and F). Due to the high level of agreement of the predicted mechanism across multiple individuals, these linkages anticipate a high likelihood of true regulatory associations. This highlights the potential of our strategy to enrich for functional ncRNAs as more individuals are sequenced.

### GWAS integration permits assignment of non-coding risk variants to pcGene targets

To explore the functional relevance of the predicted regulatory relationships, we explored genome-wide significant SNPs (*P* < 5 × 10^−8^; *n* = 89 469) from the NHGRI-EBI GWAS Catalog [35] and noticed that GWAS variants overlap the linked ncRNA and/or the associated pcGene locus (Fig. 5E and G). Of the 324 linked ncRNAs, 28.09% (*n* = 91) overlapped at least one non-coding GWAS-SNP (Fig. 5E and G), enabling the assignment of trait-associated variants to putative target genes. Conversely, 50.11% of the 457 linked pcGenes harbored one or more GWAS variants across different tissues, suggesting that our framework can also assign phenotypic traits to linked ncRNAs indirectly (Fig. 5F and G). Overall, our approach assigned 250 non-coding GWAS variants overlapping ncRNAs to putative protein-coding targets and identified 977 GWAS variants located within linked pcGenes (Fig. 5G). All ncRNA-mRNA ASE events and the integrated GWAS overlap have been made publicly available to enable further exploration (see “Data availability” section, Supplementary Tables S15 and S16). These results provide valuable insights into the disease-related landscape of regulatory ncRNAs and associated risk variants in the relevant tissues. With a growing pool of GWAS data and individuals being sequenced, our strategy has great potential to identify the functional targets of risk variants located in the non-coding genome and uncover upstream regulatory mechanisms of trait-associated coding genes.

## Discussion

A significant proportion of disease variants are located in ncRNA loci, which serve as important regulators of the genome [8, 52]. Thus, linking ncRNA loci to their targets will provide critical insights for a better understanding of complex diseases. Connecting ncRNAs to their target genes presents significant challenges, primarily due to their dynamic functionality across temporal and spatial variations. This includes diverse developmental stages, tissues and cell types, and responses to environmental cues [10, 53]. To address this challenge, we used ASE, which avoids the dynamic nature by comparing allelic expression levels within identical environments. To date, numerous studies in a wide range of species, including humans, mice, plants, and yeast, have used ASE to identify the contribution of regulatory variants to gene expression [54]. However, instead of focusing on the individual effects of variants, our strategy identifies co-occurring ASE loci, providing insights beyond variant-specific effects. Given the rarity of ASE events, our discovery of significant clustering of allele-specific ncRNAs around allele-specific pcGenes, along with a high validation rate, underscores the genuine co-regulatory interplay between allele-specific ncRNAs and neighboring allelic pcGenes. Using this concept, our study identified 397 mice and 2291 human ncRNA–mRNA ASE events and their regulatory mode-of-action. This extensive resource in mice and humans serves as an ideal starting point for the community to select candidates for further investigation and validation.

Given that our approach involves comparing alleles within the same cellular environment, the predicted gene regulation of linkages is limited to *cis*-effects, which we define as regulatory interactions between loci located on the same chromosome. This approach enabled us to determine whether an ncRNA has a repressive or activating regulatory effect on nearby targets. It should be noted that the identified ASE linkages are based on statistical correlations and do not necessarily imply causation, as ASE patterns may result from co-regulation by shared enhancers or other epigenetic influences [23]. Although our combined H3K27ac and transcriptomic analysis points to an RNA-mediated mechanism, we cannot rule out the involvement of DNA regulatory elements within the ncRNA locus, the transcription process itself, or promoters acting as enhancers in the independent modulation of adjacent gene expression [52].

Expanding our scope to humans, our results showed that the majority of linkages were identified in single individuals, consistent with the reported assumption that most ASE is driven by genetic variation [21, 24]. We evaluated the mechanism assignment on the imprinted *MEG3*/*DLK1* loci and observed a correct prediction of 79.50%. The remaining misassignment rate is likely due to phasing inaccuracies that increase with distance. The outbred nature of the human population results in a distinctive allele-specific landscape for each individual, shaped by their unique set of genetic variants. This inherent diversity provides the opportunity to uncover novel associations between ncRNAs and their targets with each individual. It is important to note that the ncRNA mechanisms and targets identified by these individual-specific associations are expected to be common across individuals, but can only be detected in individuals with a genotype that leads to allelic bias. In addition, we identified a subset of linkages that we defined as high-confidence examples based on their ability to replicate across individuals with common mechanisms-of-action. Importantly, by incorporating more and more individuals from genetically diverse cohorts in future studies, it will be possible to not only uncover novel *cis*-regulatory interactions but also naturally validate our predictions by observing consistent regulatory mechanisms across the population.

Finally, by integrating GWAS data, we were able to link non-coding variants to their targets and uncover upstream regulatory mechanisms of trait-associated coding genes. The continued accumulation of individual sequencing data and non-coding risk variants provides great potential to increase this fraction in the future. It is also important to note that another large proportion of non-coding GWAS variants are located in regulatory DNA elements, such as enhancers [2]. Notably, our strategy enables the integration of these DNA regulatory elements, offering a framework to link associated disease variants to their nearby targets. In conclusion, our approach provides a novel framework to uncover the targets and mode-of-action of the non-coding genome. With the future availability of more extensive individual sequencing data and non-coding risk variants, our strategy will illuminate the vast landscape of functional *cis*-acting elements, thereby decoding the intricate complexity of the non-coding genome and its role in complex diseases.

## Supplementary Material

gkaf912_Supplemental_Files

## Data Availability

All ncRNA-mRNA ASE events for mice and human are provided in the Supplementary Table. Further, we provide access to all linkages via the Integrative Genomics Viewer to present the target and mechanism predictions for each ncRNA for the community to explore. This resource can be accessed at https://github.com/AndergassenLab/Allelome.LINK/. The source code for Allelome.PRO2 and Allelome.LINK is available at the same GitHub page and Zenodo at https://doi.org/10.5281/zenodo.16949498. Sequence data and alignments have been submitted to the Gene Expression Omnibus (GEO) database under accession code GSE269513.
