# Supplementary material for: Allele-specific genomics decodes gene targets and mechanisms of the non-coding genome
Source: Nucleic Acids Res. 2025 Oct 21;53(19):gkaf912. doi: 10.1093/nar/gkaf912 (PMC12539623; doi:10.1093/nar/gkaf912)
Supplement: gkaf912_Supplemental_Files [file gkaf912_supplemental_files.zip › 03_SUPPLEMENTARY_FIGURES_R1.pdf]

Allele-specific genomics decodes gene targets and mechanisms of the non-coding genome

AUTHORS

Tim P. Hasenbein[1,2], Sarah Hoelzl[1,2], Stefan Engelhardt[1,2], and Daniel Andergassen[1,2*]

[1] Institute of Pharmacology and Toxicology, Technical University Munich (TUM), Munich, Germany
[2] DZHK (German Centre for Cardiovascular Research), Partner Site Munich Heart Alliance, Munich, Germany

* To whom correspondence should be addressed. Email: daniel.andergassen@tum.de

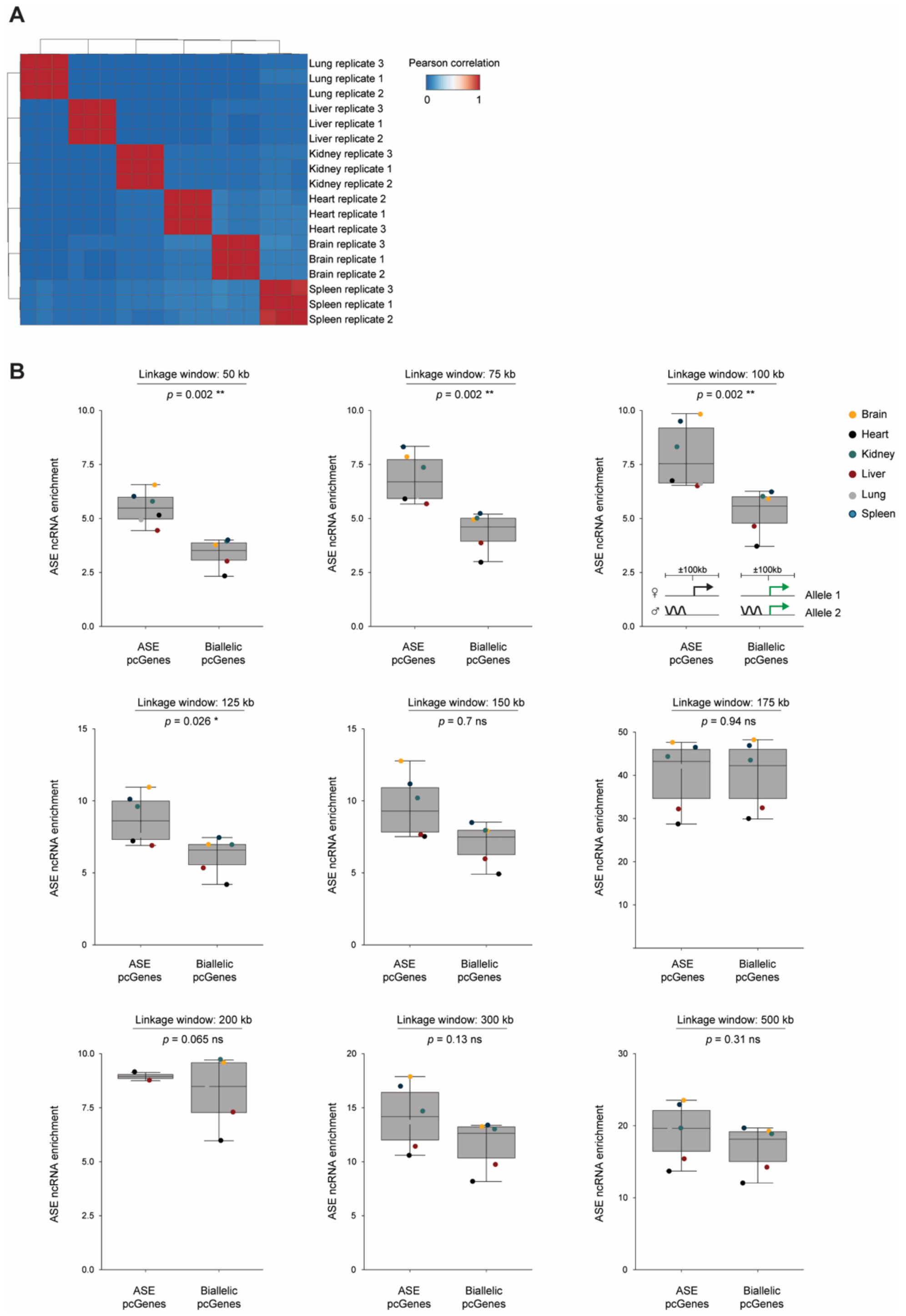

Supplementary Fig S1. Transcriptome and allele-specific enrichment analysis across the major mouse organs.

(**a**) Heatmap showing unsupervised clustering of the Pearson correlation calculated on transcripts per million values for adult tissue samples from lung, liver, kidney, heart, brain and spleen samples (n = 18).

(**b**) Box plots illustrating the percentage of allele-specific ncRNAs nearby allele-specific and biallelic protein-coding genes (pcGenes) for various window sizes ranging from 50 – 500kb. Boundaries of boxes indicate the interquartile range around the median. Whiskers range from maximum to minimum values. Wilcoxon tests were used to compare the allele-specific ncRNA enrichment around allele-specific and biallelic genes (p = 0.002). Asterisks indicate the significance level. ns: non-significant.

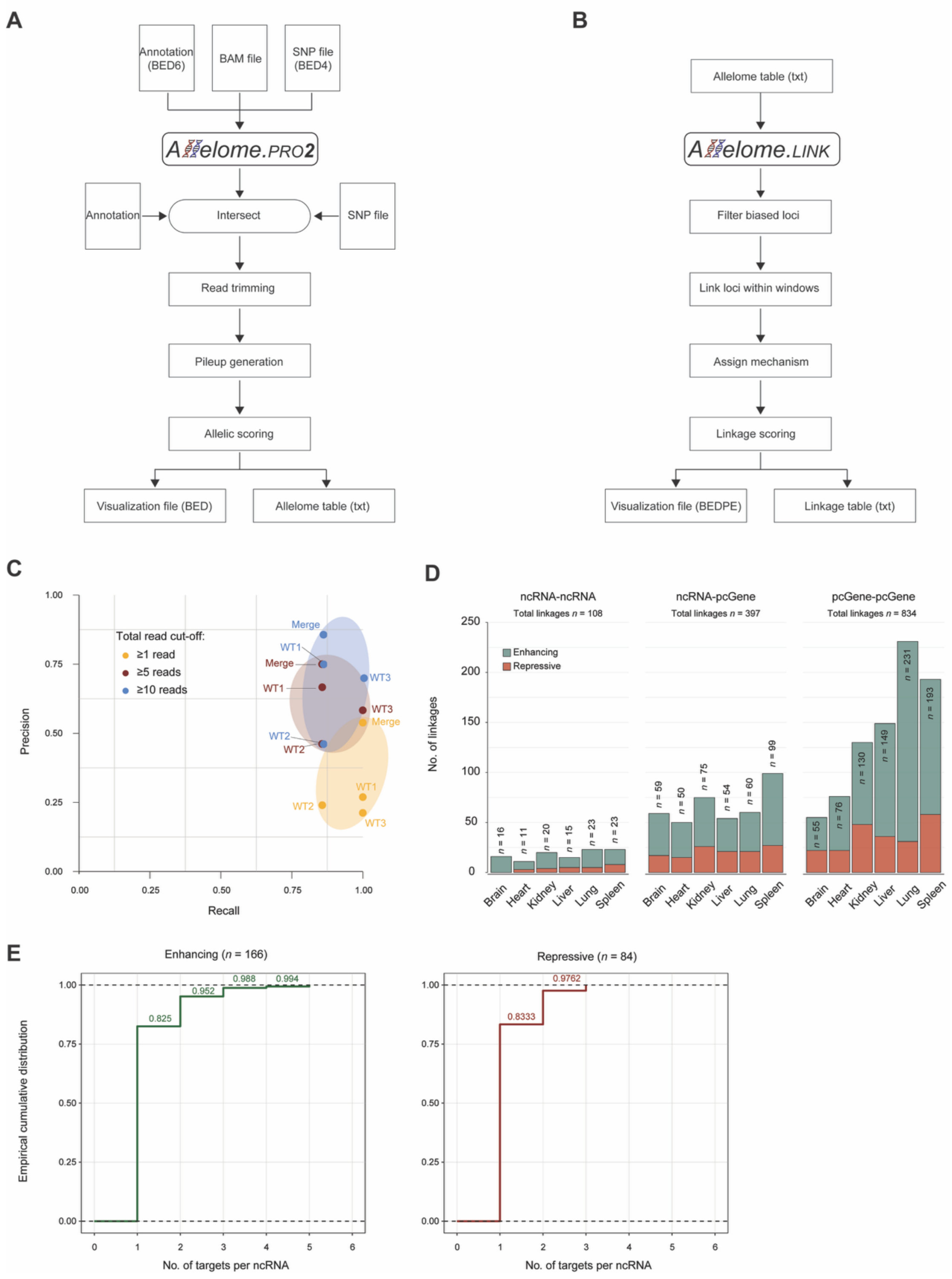

Supplementary Figure S2. Overview of the Allelome.PRO2/LINK approach and experimental validation.
(**A**) Overview of the Allelome.PRO2 pipeline. Allelome.PRO2 requires three input files: an annotation file, a BAM file, and a SNP file. Allelome.PRO2, first intersects the annotation with the SNP file. Then, read trimming is performed to ensure that each read overlaps only one SNP. Sequencing reads with their corresponding SNPs are stored in a pileup file, which is used for allelic scoring. As output, Allelome.PRO2 generates a visualization file and a table containing the allelic ratio profiles of the sequencing reads.

(**B**) Overview of the Allelome.LINK pipeline. Allelome.LINK uses the locus_table.txt output from Allelome.PRO2 as input. Biased loci are filtered according to a specified allelic ratio cutoffs. Loci with sufficient read coverage are then linked within predefined window sizes. The mechanism for these linkages is determined based on allelic correlation or anti-correlation. A linkage score is calculated for each identified interaction. The output includes a visualization file and a linkage table containing the predicted linkages.
(**C**) Precision-recall plot showing the performance of Allelome.LINK for predicting *Airn*-associated gene targets across three biological replicates (WT1, WT2, WT3) and the merged dataset, using different minimum read coverage thresholds: ≥1 read (yellow), ≥5 reads (dark red), and ≥10 reads (blue).
(**D**) Bar plots show the number of allele-specific linkages identified per organ, grouped by interaction type: ncRNA-ncRNA (left), ncRNA–pcGene (middle), and pcGene-pcGene (right). Each bar is subdivided by mechanism, with enhancing (green) and repressive (red) interactions. The total number of linkages for each interaction type is indicated.
(**E**) Empirical cumulative distribution functions showing the number of enhancing (green) and repressive (red) linkages per ncRNA after removing duplicated linkages across tissues. Colored numbers along the curves indicate the fraction of ncRNAs with the corresponding number of targets.

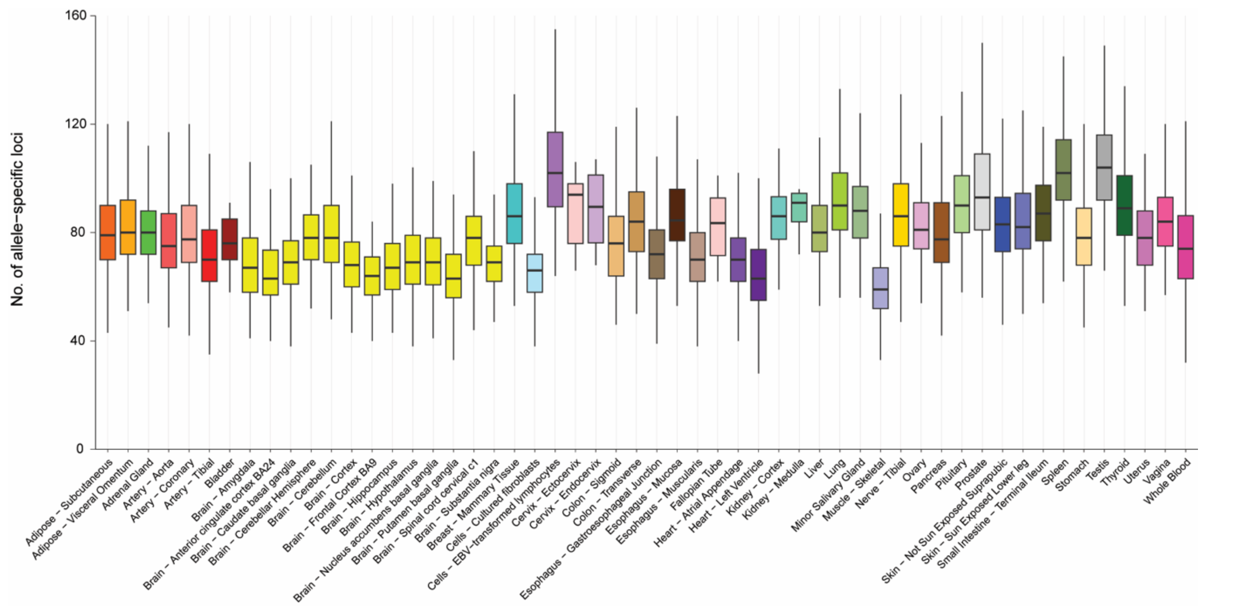

Supplementary Figure S3. Allele-specific expression across tissues in humans from the GTEx consortium. Boxplots show the number of allele-specific loci for individuals from each of the 54 different human tissues obtained from the GTEx database. Boundaries of boxes indicate the interquartile range around the median. Whiskers range from maximum to minimum values. Colors represent the different tissue samples.

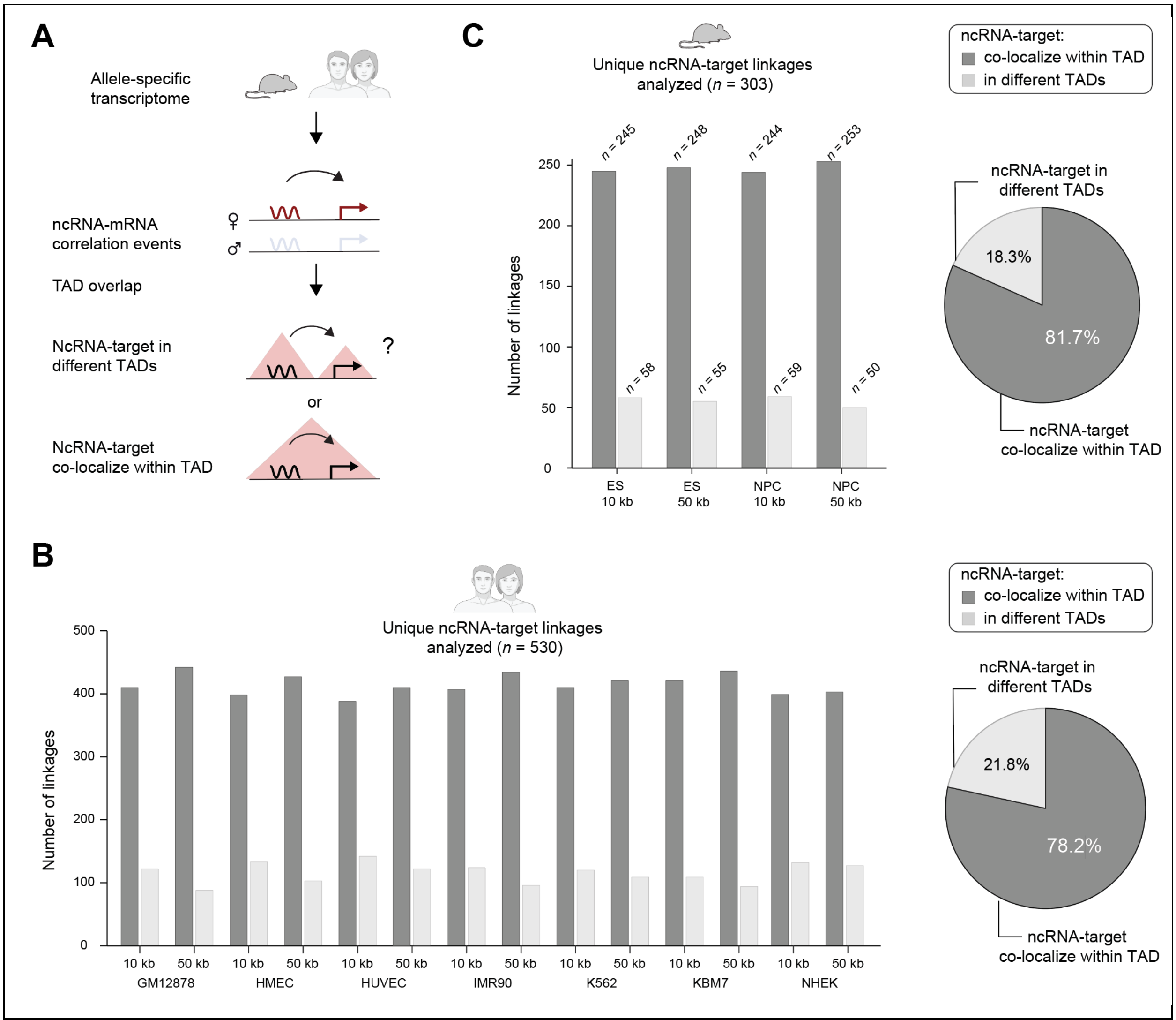

Supplementary Fig. S4: The majority of ncRNA-target linkages co-localize within the same topologically associating domain in mouse and human.
(**A**) Schematic overview of the analysis pipeline. Allele-specific transcriptome data of mice and human were used to identify ncRNA-targets correlation events, followed by the assessment of whether the linked ncRNA-targets colocalize within the same or separate TADs.
(**B**) Number of total human ncRNA-target linkages (*n* = 430) that co-localize within the same TAD (dark gray bars) or are found in separate TADs (light gray bars) across 7 human cell types (GM12878, HMEC, HUVEC, IMR90, K562, KBM7, NHEK) using 10kb and 50kb windows. The pie chart shows the average proportion of ncRNA-mRNA linkages that co-localize within the same TAD or span different TADs across the human datasets. TADs were obtained from (1)
(**C**) Number of total mice ncRNA-target linkages (*n* = 303) that co-localize within the same TAD (dark gray bars) or are found in separate TADs (light gray bars) in embryonic stem (ES) cells and neural progenitor cells (NPCs), using a 10kb or 50kb correlation window. A pie chart shows the overall proportion of ncRNAtarget correlation events that co-localize within the same TAD versus those in different TADs.

1. Liu, T., Porter, J., Zhao, C., Zhu, H., Wang, N., Sun, Z., Mo, Y.Y. and Wang, Z. (2019) TADKB: Family classification and a knowledge base of topologically associating domains. *BMC Genomics*, **20**, 217.
